# Is Platinum Present in Blood and Urine from Treatment Givers during Hyperthermic Intraperitoneal Chemotherapy?

**DOI:** 10.1155/2010/649719

**Published:** 2010-07-01

**Authors:** Sara Näslund Andréasson, Helena Anundi, Sig-Britt Thorén, Hans Ehrsson, Haile Mahteme

**Affiliations:** ^1^Department of Main Operating Theatres, Uppsala University Hospital, 751 85 Uppsala, Sweden; ^2^Department of Occupational and Environmental Medicine, Uppsala University Hospital, 751 85 Uppsala, Sweden; ^3^Department of Thoracic Surgery, Uppsala University Hospital, 751 85 Uppsala, Sweden; ^4^Department of Oncology and Pathology, Karolinska Institute and Karolinska Pharmacy, Karolinska University Hospital, 141 86 Stockholm, Sweden; ^5^Department of Surgery, Uppsala University Hospital, 751 85 Uppsala, Sweden

## Abstract

*Background*. In selected patients with peritoneal carcinomatosis (PC) originating from colorectal cancer (CRC) the high dosage of oxaliplatin (460 mg/m^2^) is recommended for hyperthermic intraperitoneal chemotherapy (HIPEC), which may be a health risk to those administering the drug. The aim of this study was to determine the risk of platinum (Pt) exposure for the two main people handling and administering the cytotoxic agent during HIPEC. *Methods*. Samples of blood and urine were collected from one male surgeon and one female perfusionist during oxaliplatin-based HIPEC treatment with open abdomen coliseum technique on six consecutive patients with PC from CRC. *Results*. All blood samples analysed were below the detection limit of <0.05 nmol/L Pt, and the urine samples were all below the detection limit of <0.03 nmol/L Pt. *Conclusions*. There appears to be little or no risk of Pt exposure during HIPEC when the recommended protective garment is used and the safety considerations are followed.

## 1. Introduction

Peritoneal carcinomatosis (PC) from colorectal cancer (CRC) is often associated with poor prognosis [1–3]. However, growing evidence indicates cytoreductive surgery (CRS) combined with hyperthermic intraperitoneal chemotherapy (HIPEC) is an effective treatment for patients in this category [4–6]. 

Oxaliplatin is a third generation platinum (Pt) complex used intravenously, at the dose of 85–100 mg/m^2^ [7, 8], to treat advanced CRC. With HIPEC, a higher oxaliplatin dose of 460 mg/m^2^ is recommended [9], and with this dosage, a 65%–75% overall survival rate of two years and an estimated five-year survival rate of 40% are reported [5, 10]. 

The use of oxaliplatin in the HIPEC setting may be a health risk for those administering the cytotoxic agent, as the heated cytotoxic agents may vaporise and become inhaled by health personnel [11]; thus, the guidelines should be followed to minimise exposure [12]. Although insignificant amounts of oxaliplatin are vaporised during HIPEC [13], the occurrence of Pt in surgeons and perfusionists exposed to oxaliplatin has not been investigated. 

The aim of this study was to determine, through blood and urine sampling, the risk of Pt exposure for the two main people handling and administering the cytotoxic agent during HIPEC.

## 2. Material and Methods

In 2008, six consecutive patients (4 men and 2 women, mean age 54.2 years (range 43–65)) with PC from CRC, with a mean peritoneal cancer index score 16.3 (range 7–32), underwent CRS and oxaliplatin-based HIPEC treatment at the University Hospital, Uppsala, Sweden. During these treatments, blood and urine samples were taken from one surgeon and one perfusionist to determine any presence of Pt, and these analyses are the basis of this report. The regional ethics committees approved the study.

### 2.1. HIPEC

HIPEC was performed by the open abdomen coliseum technique, as described earlier [14–16]. A thick plastic sheet was thoroughly sutured around the skin edges of the incision and a sterile tube system created a closed circuit for the heated chemotherapy agent to circulate. A smoke-evacuating device (Smoke Plume Evacuation System, ERBE IES 2, 2.1 m, Art nr 2-20321-012, Tübingen, Germany) with a diameter of 22 mm was connected to a suction generator (ERBE IES 2, Type nr 10321-000, App nr C-2046, Elektromedizin, Tübingen, Germany) and placed on the caudal part of the self-retaining retractor. The hose pointed towards a minimal incision in the plastic sheet where the surgeon could pass his arm and hand into the abdominal cavity to distribute the heated oxaliplatin solution. As a complement, on the opposite side of the smoke-evacuating device, an evacuation device for the anaesthetic gas was placed on the anaesthesia screen, also pointing towards the incision in the plastic sheet. 

The patients received a dose of 460-mg/m^2^ oxaliplatin (Table 1) and a concomitant intravenous (iv) 5-fluorouracil (500 mg/m^2^) plus iv leucovorin (60 mg/m^2^). The body surface area determined the volume of the electrolyte-free glucose (50 mg/mL) carrier solution. The roller-pump was set to a flow of 2 L/min, and the heat exchanger was set to heat the in-flowing oxaliplatin to 45–46°C. The mean temperature in the abdominal cavity during HIPEC was 42.2°C (range 39.7–43.4°C). The perfusion lasted for 30 minutes, as described by Elias [9]. The oxaliplatin was evacuated directly after complete perfusion. 

For HIPEC, the main two people compelled to stay in the operating room are the surgeon, who is responsible for the treatment and distributes the cytotoxic agent in the patient's abdomen by hand, and the perfusionist, who circulates the chemotherapy via a heat exchanger with the help of a roller-pump. Throughout the HIPEC, the surgeon (sterile) is placed in the surgical field, and the perfusionist (nonsterile) stands approximately 1–1.5 meters from the surgical field.

### 2.2. Protective Garment

The surgeon was dressed in a protective barrier sterile garment, wearing a high power filtration mask (3M Health Care Respirator, 1883, EN 149:2001, FFP3, 3M Svenska AB, avd. Hälsovard, Sollentuna, Sweden) and eye protection, as described by González-Bayón et al. [14]. A double pair of latex gloves (Biogel Eclipse Indicator, Ref 60775, Mölnlycke Healthcare, Gothenburg, Sweden) was used together with an extra long latex glove (WRP Dermagrip, Elbow Length Procedure Gloves, Reorder #: D3985-15, Vienna, Austria) on the arm penetrating the plastic sheet to circulate the cytotoxic agent. The perfusionist wore a nonsterile protective garment, a high power filtration mask, and nonsterile gloves specially made for toxic agents (Lirtin—Extra Long, 100% Nitrile—Non Latex. Exam gloves, Powder free. Art.no: 21-0247. SELFATRADE, Spånga, Sweden).

### 2.3. Sampling

Samples of blood and urine were collected from one male surgeon and one female perfusionist during all six treatment occasions. Both surgeon and perfusionist were in excellent health, and neither of them took any medication at the time of the study. The surgeon was 179 cm and weighed 70 kg, the perfusionist was 172 cm and weighed 67 kg. They had no history of liver- or kidney problems/disease. Creatinine levels were 65 *μ*mol/L (surgeon) and 66 *μ*mol/L (perfusionist). 

On each of the six treatment occasions, three samples of blood per person were drawn: one sample five minutes before, one sample 15 minutes during the 30-minute HIPEC-procedure, and the last sample 45 minutes after the treatment ended. Three urine samples per person and procedure were collected: one just before HIPEC; one approximately two hours after completed treatment; one 12–15 hours after HIPEC. From each of the six procedures, twelve specimens were collected around and during the 30-minute procedure. 

Urine samples were collected in 50 mL plastic bottles and blood samples in 10 mL heparin containing tubes. After collection, the samples were stored at 4°C, before being sent by mail for analysis at ALS Scandinavia AB (Luleå, Sweden). Whole blood samples (1 mL) were microwave-digested (1200 W 80%, ramp 10 minutes up to 180°C, 180°C for 50 minutes) in 1 mL 65% nitric acid in Teflon vessels. The urine samples (0.5 mL) were diluted with 8.5 mL Millipore water and 1 mL nitric acid before analysis. Platinum in the prepared samples was measured by inductively coupled plasma-sector field mass spectrometry (ICP-SFMS): the method was modified from EPA method 200.7 and 200.8 [17, 18]. The detection limit for Pt in blood was 0.05 nmol/L and for Pt in urine 0.01 nmol/L.

## 3. Results

During this study, 18 samples of blood and 18 samples of urine were collected per person (a total amount of 36 samples of blood and 36 samples of urine were analysed). Analyses from blood samples taken five minutes before, 15 minutes during the 30-minute HIPEC-procedure, and 45 minutes after the treatment revealed no detectable levels of Pt. Furthermore, analyses of urine samples taken just before HIPEC, approximately two hours after completed treatment, and 12–15 hours after HIPEC revealed no detectable levels of Pt. 

The 36 blood samples were all below the detection limit of <0.05 nmol/L, and the 36 urine samples were all below the detection limit of <0.01 *μ*g/L.

## 4. Discussion

No detectable signs of Pt in the blood or urine of either the surgeon, who was most at risk by being closest to the cytotoxic agent, or the perfusionist during six 30-minute HIPEC-perfusions of oxaliplatin were detected. This might be due to the specific high power filtration masks used during HIPEC and that the open abdomen was covered with a plastic sheet. As the surgeon was exposed to direct contact with oxaliplatin while stirring by hand in the peritoneal cavity, this could indicate the importance of wearing three layers of gloves, where one must cover the arm to the elbow [14]. 

The analysis of Pt was in whole blood, as about 40% of Pt is distributed in the red blood cells [19]. Urine was sampled as renal elimination is the major excretion pathway and about 50% of Pt is excreted in urine [20]. The analytical technique used has an extremely low detection limit (<0.05 nmol/L), and despite optimal sampling, no Pt was detected. The oxaliplatin dose given to the patients in this study was the same as in a previous study [21], which determined a high concentration of oxaliplatin in the patients' blood. As oxaliplatin was present in the blood of the patients in the earlier study, the dose was considered representative for this study, and no positive control was needed. 

A study by Guerbet et al. [13] (2007) uses oxaliplatin in three different concentrations (230, 460, and 920 mg/L), which were heated to 41, 43, and 45°C, during 30, 60, and 90 minutes. The purpose was to determine, by a coupled mass spectrometry (ICP-MS), if oxaliplatin/Pt vaporised, and as a result, can be a health hazard to surgeons and theatre staff during HIPEC. No significant quantities of Pt in the air were detected. Although the present study was comparable to the study by Guerbert et al. [13], it was more homogeneous, as dosages of oxaliplatin, the in-flow temperature of oxaliplatin, and duration of the treatment were the same throughout all samplings. 

A similar study with Mitomycin C [22] analyses samples of air from the operating room and urine from a surgeon and a perfusionist during 10 procedures. No detectable levels of the cytotoxic agent were present in any of the samples. As the open technique was used, the authors [22] conclude Mitomycin C present in the aerosols is suctioned away from the surgical field during intraoperative chemotherapy perfusion. This implies that the plastic sheet plays an important role in the operating room environment. 

Occupational exposure of Pt-based cytotoxtic agents in blood and urine has been investigated. Analyses of Pt in the blood and urine of pharmacists, and graduate and staff nurses at three different hospitals with various facilities and routines on how to handle cytotoxic agents, reveals no increased Pt levels in the blood of pharmacists, but increased levels in nurses, more so in the staff nurses caring for the patients [23]. Graduate nurses who handled the drugs were less exposed. In hospitals with better routines and facilities, exposure was lower. 

Heating the intraperitoneal cytotoxtic agent is important in treating PC of CRC. The cytotoxic agent in HIPEC is considered effective and responsible for the destruction of cancer cells, but hyperthermia might induce cytotoxicity and apoptosis [24, 25]. Hypertherm chemotherapy appears to penetrate tumour cells more efficiently and decreases vascularity [15]. However, results vary depending on different tumour cells [24]. Tests on the cytotoxic effect of hypertherm oxaliplatin indicate that the potency of the drug intensifies as it is heated to 41°C [26].

Cytotoxic agents are a risk and there are clear directions from the Occupational Safety and Health Administration (OSHA) and the National Institute for Occupational Safety and Health (NIOSH) on handling and administration [12, 27]. When heating these agents, as in HIPEC, aerosols are likely to be inhaled by the personnel administering the treatment [11, 13]. This is the reason HIPEC safety considerations are documented [11, 14, 28] and should be followed at all times. The risk of exposure needs to be determined in order for this treatment to be fully acceptable, that is the outcome for the patient against the exposure of the treatment staff to the vaporisation of oxaliplatin. 

In conclusion, little or no risk of Pt exposure during HIPEC is apparent when the recommended protective garments are used and safety considerations are followed.

## Figures and Tables

**Table 1:** Oxaliplatin perfusion.

Patient (sex)/diagnosis	Weight (kg)	Length (cm)	Surface area (m^2^)	Dose of oxaliplatin during HIPEC (mg)
Female/CRC with PC	48	158	1.47	600
Male/CRC with PC	96	178	2.14	960
Male/CRC with PC	87	185	2.11	960
Male/CRC with PC	82	173	1.96	890
Male/CRC with PC	80	170	1.91	870
Female/CRC with PC	72	164	1.78	380

*CRC*: Colorectal cancer; *PC*: Peritoneal carcinomatosis; *HIPEC: *hyperthermic intraperitoneal chemotherapy.
